# ARTS as a cardiometabolic biomarker to bridge gaps in dementia diagnostics and therapeutics

**DOI:** 10.1002/alz.70811

**Published:** 2025-10-21

**Authors:** Timothy Daly, Bruno P. Imbimbo

**Affiliations:** ^1^ Bioethics Program FLACSO Argentina Buenos Aires Argentina; ^2^ Research & Development Chiesi Farmaceutici Parma Italy

1

Cerebral arteriolosclerosis is a form of small vessel disease characterized by the hardening and thickening of deep arterioles of the brain, and is commonly observed in conditions such as hypertension, diabetes, and dementia. In a diverse community sample, Fleischman et al. found that an in vivo magnetic resonance imaging‐based classifier of arteriolosclerosis (shortened to ARTS), based on demographic data, white matter hyperintensity burden, and measures of diffusion tensor imaging fractional anisotropy, was a reliable marker for risk of mild cognitive impairment, dementia, and stroke.^1^ Arteriolosclerosis is a pathogenic mechanism that serves not just as a marker, but also provides an upstream explanation of reductions in brain blood flow, infarcts, and downstream impacts on cognitive impairment.^2^ In dementia research and clinical practice, ARTS could thus provide a polyvalent biomarker of cardiometabolic contributions to cognitive impairment due to underlying conditions such as hypertension and diabetes,^3^ for example, alongside neurodegenerative biomarkers of amyloid beta (Aβ) and tau of Alzheimer's disease (AD) in both diagnostics and therapeutics.

Concerning diagnostics, in the absence of neurodegenerative pathologies such as AD, ARTS could be used to confirm the diagnosis of vascular dementia in cognitively impaired persons, which currently relies on gross measures of infarcts, white matter damage, and atrophy.^4^ In the presence of AD neuropathology, ARTS could provide a quantifiable measure of cardiometabolic comorbidities that independently contribute to cognitive decline.^5^ Indeed, Section 7.2 of the Alzheimer's Association 2024 revised criteria for the diagnosis of AD^6^ discusses “Biomarkers of common non‐AD copathologies” and recognizes that while there are multiple neuroimaging markers of cerebrovascular disease, none of them are specific, and there is no unified summary measure for vascular brain injury, creating diagnostic ambiguity. ARTS is a promising alternative to current imaging‐based markers of cerebrovascular disease that provides a quantifiable measure that is also specific, mechanistic, and clinically relevant.

As a therapeutic example of ARTS’ application, take the example of autosomal dominant AD, for which Müller et al. found that World Health Organization–defined high levels of physical activity were associated with up to 15 years longer cognitive function, but not due to reduced global Aβ burden.^7^ Cardiometabolic biomarkers such as ARTS could thus be used complementarily alongside neurodegenerative biomarkers to provide a mechanistic explanation of the positive impacts of physical activity on cognitive function. In clinical trials, such as those testing the therapeutic value of cardioprotective glucose‐lowering drugs such as metformin^8^ and semaglutide^9^ in AD, a similar logic could be used: Cummings’ criterion of correlation between clinical efficacy and a biomarker in the same trial^10^ could be used to establish that putative treatments are either disease modifying when ARTS is specific (e.g., ARTS in vascular dementia)—or meaningfully reduce comorbidities when ARTS is an independent contributor to worsened cognition (e.g., ARTS in AD).

To qualify as a reliable biomarker for detecting cardiometabolic contributions to cognitive decline, ARTS should be further developed to meet the core US Food and Drug Administration criteria for approval of a diagnostic test: analytical performance, clinical validation, and clinical utility. This would require demonstrating robust and reproducible measurement methods for ARTS, confirming its accuracy to detect *post mortem* arteriolosclerosis in well‐characterized cohorts as suggested by the authors (p. 12,^1^), and building the evidence base that its use would inform clinical decision making and/or improve patient outcomes.

## CONFLICT OF INTEREST STATEMENT

Dr. Timothy Daly has no conflicts of interest to declare. Dr. Bruno P. Imbimbo is an employee at Chiesi Farmaceutici. He is listed as an inventor in a number of Chiesi Farmaceutici's patents for anti‐Alzheimer's disease drugs. All author disclosures are in the supporting information.

## Supporting information



Supporting Information
